# Democratizing clinical-genomic data: How federated platforms can promote benefits sharing in genomics

**DOI:** 10.3389/fgene.2022.1045450

**Published:** 2023-01-10

**Authors:** Maria Alvarellos, Hadley E. Sheppard, Ingrid Knarston, Craig Davison, Nathaniel Raine, Thorben Seeger, Pablo Prieto Barja, Maria Chatzou Dunford

**Affiliations:** Lifebit Biotech Limited, London, United Kingdom

**Keywords:** federation, genomics, cloud computing, trusted research environment, clinical genomics

## Abstract

Since the first sequencing of the human genome, associated sequencing costs have dramatically lowered, leading to an explosion of genomic data. This valuable data should in theory be of huge benefit to the global community, although unfortunately the benefits of these advances have not been widely distributed. Much of today’s clinical-genomic data is siloed and inaccessible in adherence with strict governance and privacy policies, with more than 97% of hospital data going unused, according to one reference. Despite these challenges, there are promising efforts to make clinical-genomic data accessible and useful without compromising security. Specifically, federated data platforms are emerging as key resources to facilitate secure data sharing without having to physically move the data from outside of its organizational or jurisdictional boundaries. In this perspective, we summarize the overarching progress in establishing federated data platforms, and highlight critical considerations on how they should be managed to ensure patient and public trust. These platforms are enabling global collaboration and improving representation of underrepresented groups, since sequencing efforts have not prioritized diverse population representation until recently. Federated data platforms, when combined with advances in no-code technology, can be accessible to the diverse end-users that make up the genomics workforce, and we discuss potential strategies to develop sustainable business models so that the platforms can continue to enable research long term. Although these platforms must be carefully managed to ensure appropriate and ethical use, they are democratizing access and insights to clinical-genomic data that will progress research and enable impactful therapeutic findings.

## 1 Introduction

Genomic technologies are rapidly advancing the integration of genomics into clinical care, with evidence demonstrating their role in disease diagnosis, drug discovery and targeted therapeutics (Green et al., 2020; Atutornu et al., 2022; Borle et al., 2022). Digital health records, next generation sequencing, and artificial intelligence (AI) are also leading to an explosion of health data (Asiimwe et al., 2021). As observed within research, increased sample size improves the potential for discovery: genome wide association studies (GWAS) are prime examples, where it has been shown that a 10-fold increase in sample size can lead to a 100-fold increase in identified loci with significant disease associations (Visscher et al., 2017). Due to their sensitive nature, clinical, phenotypic and omics datasets are primarily distributed and stored in siloed, inaccessible locations (Asiimwe et al., 2021; Garden, 2021); in a stark example, the World Economic Forum estimates that 97% of all hospital data goes untouched^1^.

Between nations there exists strict national regulatory frameworks governing the movement of patient data and limiting transfer between national jurisdictions, which poses a significant barrier when trying to access international datasets (Mitchell et al., 2020). Adding to the complexity of using such data to derive meaningful insights, it is well-recognized and unfortunate that most genomic data does not represent diverse populations. A lack of diverse representation in clinical-genomic datasets ultimately limits the clinical utility of genetic findings as low sample sizes are insufficiently powered to identify disease-causing variants for specific populations (Atutornu et al., 2022; Lee et al., 2022).

Despite these challenges, there are ongoing efforts to increase the useability of clinical, phenotypic and multi-omic data for diagnosing and treating disease. Federated data platforms are emerging as means to achieve data accessibility, useability and security while adhering to governance and privacy regulations (Saunders et al., 2019; Blomberg and Lauer, 2020; Nik-Zainal et al., 2022). In this perspective, we explore how federated models for data access and analysis and end-to-end platforms can help to facilitate genomic benefits sharing; democratizing access to global data assets and insights facilitates the linking of diverse datasets to improve representation. We describe successful examples of how federation is being adopted across research and healthcare settings and discuss ongoing challenges and recommendations. Moving forward, it is imperative to build upon these technologies to ensure breakthroughs in genomic medicine for all. Safe and secure access to usable, diverse genomic data is poised to rapidly progress research and benefit patients.

## 2 Overcoming secure data sharing *via* federated platforms

### 2.1 Federated biomedical data platforms are emerging worldwide

Federation, in its simplest terms, is a software process that allows multiple databases to function as one. Federated architecture is a technological blueprint that facilitates interoperability and information sharing between autonomous, decentralized organizations. Within a federated architecture, data will remain within appropriate jurisdictional boundaries, while metadata are centralized and searchable. This is an alternative to a model in which data is moved or duplicated then centrally housed. Federated architectures of individual organizations may be connected together into a federated data platform, enabling data access and computation for users across organizations. We consider full federation to occur when both data and compute access are federated over distributed compute and databases to allow querying and joint analyses over the data (Chaterji et al., 2019). However, there also exists the potential for partial federation (I and II), when either compute access or data access are federated and compute or databases are distributed (Table 1). This is distinct from federated learning, which has tackled this problem in the context of Machine Learning (ML) in healthcare—researchers can train machine algorithms collaboratively on dispersed data, including health records, without infringing on data governance legislations (Mandl and Kohane, 2015; Stephens et al., 2015; De Fauw et al., 2018; Rieke et al., 2020; Xu et al., 2021; Pati et al., 2022).

**TABLE 1:** Levels of federation.

No federation	Partial federation I (federated learning)	Partial federation II	Full federation
**Mode of data access**	**Mode of data access**	**Mode of data access**	**Mode of data access**
• Manual access to different organizations and analysis	• Results aggregation analysis is centralized	• Federated data access, distributed databases and joint analyses	• Federated data access
• Results are aggregated and sent back to a central platform			
**Mode of compute access**	**Mode of compute access**	**Mode of compute access**	**Model of compute access**
• Centralized compute; results aggregation analysis	• Federated compute access, distributed compute	• Centralized (*via* on-demand streaming)	• Federated compute access, distributed compute and databases, joint querying over distributed data and joint analysis
**Requirements**	**Requirements**	**Requirements**	**Requirements**
• Manual intervention	• Requires a central, unified and federated platform and federated access for compute (i.e., *via* API)	• Requires a central unified and federated platform and federated access for data queries and retrieval (i.e., *via* API, database queries)	• Requires a central, unified and federated platform or cleanrooms across each network in the federation
• Containerized/portable, versioned and FAIR tools/algorithms that humans can run in different environments			
**Common use case**	**Common use case**	**Common use case**	**Common use case**
• Optimal when federated access to organizations is not permitted, e.g., a researcher downloads publically available WGS data from various sources and analyzes it together in-house	• Optimal when security and governance clearance is provided and federated linkage is permitted, e.g., the Trustworthy Federated Data Analytics consortium that enables federated data learning on disparate clinical imaging^3^	• Optimal when security and governance clearance is provided and federated linkage is permitted, e.g., ELIXIR federated data platform to connect Europe’s data sources Saunders et al. (2019)	• Optimal when security and governance clearance is provided and federated linkage is allowed (if a federated platform does not exist, a cleanroom is permitted), e.g., Lifebit federated technology bridging the trusted research environments of biobanks and national genomics initiatives to enable joint querying and analysis Nik-Zainal et al. (2022)

There is now an increasing prevalence of federated architectures to connect large-scale health data (Saunders et al., 2019; Blomberg and Lauer, 2020; Thorogood et al., 2021; Nik-Zainal et al., 2022)**.** Given the sensitive nature of health data, it cannot be physically pooled or moved for legal and regulatory reasons. This poses a challenge for researchers who rely on access and sufficient sample size to progress research. National genomic programs are increasingly adopting platforms with federated architectures to bring together distributed national datasets (Stark et al., 2019). Australian Genomics is developing a federated repository of genomic and phenotypic data to bridge the gap between its national health system and state-funded genetic services (Stark et al., 2019). In Canada, each province has its own health data privacy legislation such that data generated in each province must follow provincial governance laws. The Canadian Distributed Infrastructure for Genomics (CanDIG) platform is tackling this with a fully distributed federated data model, enabling federated querying and analysis while making sure that local data governance laws are respected (Dursi et al., 2021).

Within Europe, initiatives such as ELIXIR are linking Europe’s leading research organizations to more easily find, share and analyze data (Saunders et al., 2019; Blomberg and Lauer, 2020). ELIXIR oversees sub-initiatives including the European Genome Archive (EGA) federated networks to enable access and sharing of genomic data. ELIXIR is further participating in the Beyond 1 Million genomes (B1MG)^2^, which aims to create a network of clinical and genomic data across Europe. At a global level, the Common Infrastructure for National Cohorts in Europe, Canada, and Africa (CINECA) project is working to federate data between cohorts and across continents (Dursi et al., 2021). Through employing federated architectures, each of these initiatives allow organizations to store and manage their data locally while researchers worldwide can access the data securely.

### 2.2 Important considerations in establishing federated platforms

Federated analysis integrates with the architectures described above so that disparate data can, once securely accessed, be analyzed *in situ* across multiple sites. Establishing federated architectures requires that computing environments, systems, devices and applications within and across organizational, regional and national boundaries are all interoperable—this means overcoming the differences in multiple healthcare reporting systems, which frequently use different data models and ontologies (Mulder et al., 2017; Stark et al., 2019). Health data exchange architectures, application programming interfaces (APIs) and standards can provide a common language and set of expectations to enable interoperability between systems or devices so that authorized researchers can access and share data regardless of when or where it originates (Thorogood et al., 2021)^4^.

International initiatives have come together to tackle the issue of interoperability in federated platforms. The Global Alliance for Genomics and Health (GA4GH) sets standards to promote the international sharing of genomic and health-related data, in part by setting interoperability standards and providing open-source APIs (Thorogood et al., 2021). The GO FAIR initiative aims to implement data principles in order to make it Findable, Accessible, Interoperable and Reusable (FAIR)^5^ and the Observational Health Data Sciences (OHDSI) community is developing open-source tools to implement a common data model for combining disparate datasets^6^. The importance of widespread interoperability is increasingly reflected in the long-term strategy and funding of research institutes; in the US, the National Institutes of Health (NIH) Cloud Platform Interoperability Effort (NCPI)^7^ is establishing and implementing guidelines and technical standards for a federated data ecosystem. In the UK, the UK Research and Innovation program has recently established the Data and Analytics Research Environments UK (DARE UK) program^8^ to design and deliver a more coordinated national data research infrastructure.

While data interoperability is hugely important for federated collaborations, the data must be of a high quality. As federated data platforms lower the barrier of access to data, there must be guidelines to ensure that the data utilized in analysis is of acceptable quality to yield reliable results. For example, low-quality sequencing reads are more likely to inaccurately call variants, which can derail research efforts; within the context of precision medicine efforts, this could even lead to inaccurate diagnoses. There is now a breadth of literature highlighting the importance of quality control within sequencing analysis (NCI-NHGRI Working Group on Replication in Association Studies, 2007; Miyagawa et al., 2008; Turner et al., 2011; DeLuca et al., 2012; Ma et al., 2019), with organizations such as ENCODE^9^ offering guidelines for appropriate sequencing coverage and quality controls. As federated data platforms continue to expand, it will be important that administrative authorities designate quality thresholds for data submission, and that these should be published within the metadata catalogs for researchers.

With the ability to process immense datasets, computational resources are an important consideration. The scale of distributed multi-omics and clinical datasets available today has brought an increasing shift towards commercial cloud infrastructure. The “elastic” nature of cloud computing means researchers only pay for what they need. Further, researchers can create near identical hardware and software setups remotely, regardless of whether they are near a data center (Langmead and Nellore, 2018). Cloud computing builds capacity for state-of-the-art capabilities in encryption, firewalls and monitoring. Despite this, there is still reticence towards adopting cloud computing for genomic data in some jurisdictions; it is not fully clear how existing privacy and data protection laws apply in the genomics context and as such there is a lack of community consensus on best practices (Dove et al., 2015). Defining standards and best practices, in addition to cloud companies providing transparency of security and technology infrastructure, will be essential to build trust across the industry and enable more organizations to harness the benefits of cloud computing.

Despite these advances, any computing environment that involves sensitive patient data is not without risk (Melis et al., 2018; Nasr et al., 2018). While data remains locally for federated architectures, there is still a component that is exchanged, such as intermediate ML models or aggregated results for federated learning and analysis, respectively. With federated learning, ML models can be susceptible to security risks such as inference attacks, feature leakage and data poisoning, which can result in the leakage of unintended information about participants’ training data (Melis et al., 2018; Nasr et al., 2018). Ongoing work is needed to investigate how parameters can be further protected and how the tradeoff between the privacy and security-level versus system performance and cost should be managed (Popovic, 2017). Likewise, federated models for data access introduce unique security risks, such as when new users or code are introduced into a data controller’s computing environment (Popovic, 2017). Careful logging and auditing of platform and user activity, as well as data/code export controls (e.g., airlocks^10^), are needed to monitor these risks.

### 2.3 Federation to promote global collaboration and representation in genomic datasets

To improve disease diagnostic capabilities for the greatest number of people, larger and more diverse cohorts are needed (Zoch et al., 2021). By facilitating international cooperation *via* secure data unification, federation can support more diverse population representation in genomic datasets (Vesteghem et al., 2020; Asiimwe et al., 2021; Garden, 2021; Powell, 2021; Zoch et al., 2021; Lee et al., 2022). In academic research, initiatives like Matchmaker Exchange (MME) are demonstrating how distributed datasets of genotypes and rare phenotypes can be combined using a federated network to facilitate rapid, secure data sharing to achieve faster diagnoses (Philippakis et al., 2015; Zoch et al., 2021). The Human Heredity and Health in Africa (H3Africa) initiative is promoting intra-continental collaborations to establish a network of African-based biorepositories (Abimiku et al., 2017; Mulder et al., 2017). Already, this program is highlighting deep regional variation for disease-related risk factors and has established critical tools (genotyping arrays and reference gene panel for imputation) that support the analysis of genetic data from individuals of African descent (Mulder et al., 2017).

Despite this progress, trust remains an important issue to recruit participants, especially in historically marginalized groups. As data custodians retain control over their dataset in a federated data access model, data access agreements must be negotiated in a manner that is acceptable for research participants to engender trust, particularly in historically underrepresented groups (Thorogood et al., 2021; Lee et al., 2022).

## 3 Democratizing access to data assets and insights *via* federated platforms

### 3.1 Considerations in democratizing genomic data

A core benefit of federated data platforms is that they can democratize access to health data in a secure manner. While this brings huge potential for advancing medical research, there must be strict regulations over how data is governed and accessed that are applied at the organizational- and researcher-level, in order to engender public and participant trust.

There is a valid concern of ownership over federated data platforms—a trusted independent party, a group of institutions, or the government could theoretically assume the role. In the United Kingdom, there is currently a concerted effort across the public sector towards the establishment of a federated, research data infrastructure^11^ ^–^ [Fn fn12] ^13^. In this model, patient data is stored in trusted research environments (TREs) or “secure data environments” and federated technology is used to virtually link these environments while data stays securely at its source, always within full control of the data custodian/controller. The TRE is fully owned and governed by the data controller(s)^13^; this means there is collective ownership across the multiple healthcare providers contributing to the data source.

In line with the surge in data regulations arising across global jurisdictions^14^ ^–^ [Fn fn15] ^16^, there is an increasing prevalence of accreditation schemes to audit and certify the “owner” of data management platforms^14^ ^,^ ^17^. To guarantee ethical and secure usage of federated platforms, the safety and governance of these infrastructures must be regularly reviewed and measured against all aspects relevant to data security and governance, from implementing industry-recognised data protection frameworks^18^, standards and information security measures to compliance with local data regulations and commitments to interoperability. Access to the data within these federated platforms must be appropriately reviewed and governed by the data controllers—identifying an efficient and secure process for approving access and democratization of this data is a community-wide work in progress. Implementing such governance and regulatory bodies that regulate the use of data can help foster trust in the wider public for genomics research among the wider public and ensure data use is in the interest of both the public and participants.

### 3.2 Enabling analytics *via* no/low-code tools and end-to-end platforms

The software industry is currently shifting towards “no/low-code” tools to support a wider range of end users with and without a data science background, thus enabling full democratization of access to genomic data and the insights derived. The Galaxy Community, an initiative within ELIXIR, is one such example offering a web-based platform to facilitate computational research for a variety of “omics” types (The Galaxy Community et al., 2022). There are also resources such as DepMap^19^ that offer easy-to-use graphical user interfaces to explore cancer vulnerabilities from available chemical and genetic perturbation data using analytical and visualization tools. Together, these tools enable users of diverse backgrounds to visualize the data directly or build reproducible pipelines and complex workflows for analyses.

**FIGURE 1:**
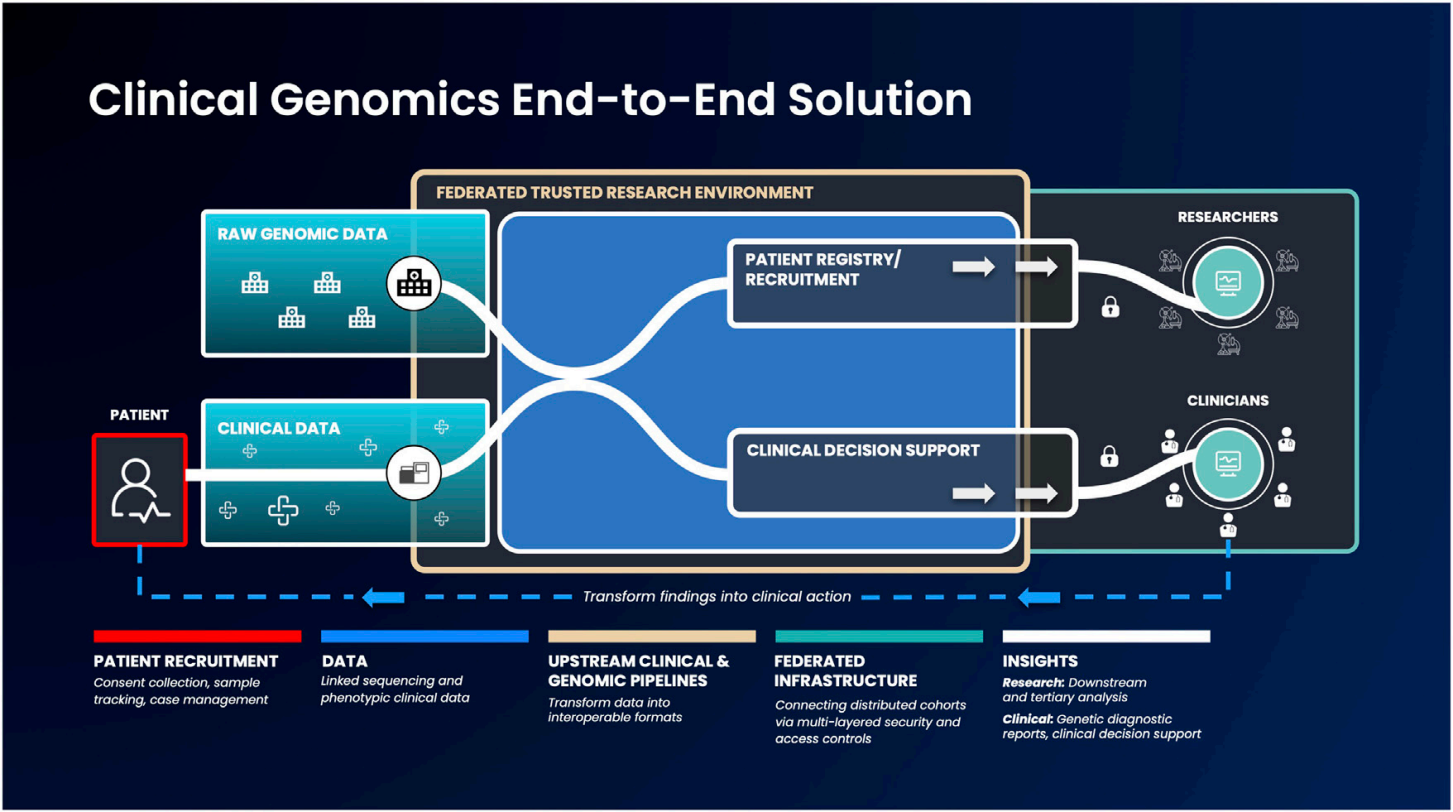
An example genomic medicine end-to-end solution that integrates federated architecture. Genomic or phenotypic clinical data is first collected and transformed into interoperable formats. Next, these data will be ingested into the federated architecture, which allows authorized users to access and combine this data with other disparate sources to build unique and valuable analysis cohorts. Strict security measures will facilitate results export to clinicians and researchers, to enable them progress therapeutic discovery and make informed clinical decisions.

While such low-/no-code tools are a huge first step, there should ideally be an end-to-end, federated solution for researchers as well as clinicians - providing the latter with the resources they require to understand their patients’ data (Kullo et al., 2013; Lau-Min et al., 2021). An end-to-end data platform, building upon the current advances of federated data architectures and capable of ingesting clinical and raw genomic data, can democratize access and accelerate the generation of clinically actionable insights. Such platforms could securely integrate between a country’s healthcare network, national genomic medicine initiatives and sequencing laboratories. When coupled with tools to enable anyone to run bioinformatic pipelines and workflows, such a platform could handle genetic services end-to-end: from patient recruitment, sample collection, sequencing, data standardization, analysis and clinical reporting (Stark et al., 2019) (Figure 1). By federating across distributed databases and systems as well as providing the necessary, easy to use tools to transform raw data into meaningful insights can bring more direct benefits to patients.

### 3.3 Ensuring the sustainability of federation for future genomics research

While many countries are increasingly, and successfully, integrating genomics into healthcare (Stark et al., 2019; Kloypan et al., 2021), it is important to note that not all learnings described here are broadly applicable. Many countries and regions are faced with rapidly shifting health priorities and challenges including low levels of government support, absence of well-funded national healthcare systems, workforce skill shortages and gaps in infrastructure (Mulder et al., 2017; Stark et al., 2019; Maxmen, 2020). Data sharing, even in a fully federated system, is associated with significant costs (Chalmers et al., 2016)^20^. The long-term sustainability of the genomics ecosystem is reliant on more sustainable solutions and secure, long-term funding, something that will only be achieved through industry-wide collaboration.

Collaboration between biobanks and the broader life sciences industry can build larger and more representative data ecosystems and open sustainable funding mechanisms for population genomics initiatives and biobanks, particularly in countries with fewer resources for research. Specifically, extending collaboration into the private sector, biobanks can accelerate growth with highly lucrative and sustainable funding. There is increasing recognition among pharmaceutical companies that diversity among the patient-participant population of clinical trials is critical given large genetic variability in drug responses that is often correlated with ancestry (Gross et al., 2022).

As the private sectors will not freely disseminate their knowledge, there is a model by which genomic initiatives and biobanks can negotiate data access agreements with pharmaceutical companies who require large and diverse patient cohorts for R&D and drug discovery pipelines (Garden, 2021; Thorogood et al., 2021). An example is that of 54 Gene, a venture capital-backed biobank based in Nigeria, which will partner with pharmaceutical companies to fund its research by charging access fees, like the UK Biobank (Maxmen, 2020). By generating stable and sustainable funding mechanisms through collaborative partnerships, biobanks and precision medicine programs can generate holistic benefits sharing at scale (Maxmen, 2020; Thorogood et al., 2021; Bedeker et al., 2022).

## 4 Discussion

Here, we have presented a perspective on the overarching progress to develop federated data platforms to enable research and genomics efforts. While there has been significant progress within national and international endeavors to provide secure access to their large-scale health data, as well as tools to empower users to derive meaningful insights, frameworks and policies guiding the genomics community on best practices for data sharing are necessary to ensure successful collaboration. These must cover critical considerations discussed in this perspective including interoperability, secure data access, cloud computing, usability, democratized data access, clinical utility, ethical considerations and sustainability of the platforms (Thorogood et al., 2021; Lee et al., 2022). Governing agencies are indeed beginning to address the complexities associated with data sharing—the World Health Organization’s recent report serving as a notable example (WHO, 2022) Within a federated ecosystem, there are roles for the private and public sectors. In this perspective, we have highlighted an opportunity for pharma to invest in biobanking and federated data platforms in order to increase their access to data, which in turn funds the platforms. Further, it may be important to consider moving forward the role of DNA testing companies in building federated networks. These companies have access to the data of millions of individuals, and it will be interesting to determine whether there are any incentives for these to join the federated data ecosystems, while also adhering to governance and privacy policies.

Finally, continued democratization of data access and analysis has the potential to broaden the reach for innovative technologies (Drake et al., 2018; Christopher et al., 2021). Future efforts to expand federated data platforms in an ethical manner will require broad coordination between non-governmental organizations, local governments, scientific researchers and industry to advocate for increased investments to build capacity and improve infrastructure^21^. Evolving federated data platforms, such as those discussed here, are already accelerating research by drawing research communities together to benefit patients. Further investment in and expansion of such sustainable platforms will continue to power research so that access and usability of data will no longer be a barrier to discovering powerful therapeutic insights.

## Data Availability

The original contributions presented in the study are included in the article/Supplementary Material, further inquiries can be directed to the corresponding author.

## Footnotes

1 World Economic Forum. 4 ways data is improving healthcare (2019). World Economic Forum. https://www.weforum.org/agenda/2019/12/four-ways-data-is-improving-healthcare/ [Accessed 27 July 2022].

2 Beyond One Million Genomes Project (2022) https://b1mg-project.eu/ [Accessed 13 December 2022].

3 Trustworthy Federated Data Analytics (TFDA) (2022). https://tfda.hmsp.center/ [Accessed 5 December 2022].

4 Healthcare Information and Management Systems Society (2020). Interoperability in Healthcare. https://www.himss.org/resources/interoperability-healthcare [Accessed 16 August 2022].

5 GO FAIR Initiative (2017). The GO FAIR Initiative. https://www.go-fair.org/go-fair-initiative/ [Accessed 16 August 2022].

6 Observational Health Data Sciences and Informatics (2022). OMOP Common Data Model. https://www.ohdsi.org/data-standardization/the-common-data-model/ [Accessed 16 August 2022].

7 NIH Cloud Platform Interoperability Effort (2022) https://datascience.nih.gov/nih-cloud-platform-interoperability-effort [Accessed 16 August 2022].

8 Data and Analytics Research Environments UK (2021). https://dareuk.org.uk/about/ [Accessed 16 August 2022].

9 ENCODE. (2022) https://www.encodeproject.org/ [Accessed 13 December 2022].

10 Importing and exporting files using the Airlock (2022). https://re-docs.genomicsengland.co.uk/airlock/#importing-and-exporting-files-using-the-airlock [Accessed 13 December 2022].

11 Genome UK: 2021 to 2022 implementation plan (2021) https://www.gov.uk/government/publications/genome-uk-2021-to-2022-implementation-plan [Accessed 16 August 2022].

12 Better, broader, safer: using health data for research and analysis (2022) https://www.gov.uk/government/publications/better-broader-safer-using-health-data-for-research-and-analysis [Accessed 16 August 2022].

13 Secure data environment for NHS health and social care data—policy guidelines (2022). https://www.gov.uk/government/publications/secure-data-environment-policy-guidelines/secure-data-environment-for-nhs-health-and-social-care-data-policy-guidelines [Accessed 13 August 2022].

14 NIH Data Management and Sharing Policy (2022). https://sharing.nih.gov/data-management-and-sharing-policy [Accessed 13 December 2022].

15 General Data Protection Regulation (2022). https://gdpr-info.eu/ [Accessed 13 December 2022].

16 CS/HB 833 — Unlawful Use of DNA (2021). https://www.flsenate.gov/Committees/billsummaries/2021/html/2543 [Accessed 13 December 2022].

17 Our Future Health opens consultation on trusted research environment accreditation process (2022). https://ourfuturehealth.org.uk/news/our-future-health-opens-consultation-on-trusted-research-environment-accreditation-process/ [Accessed 13 December 2022].

18 What is the Five Safes framework? (2022) https://ukdataservice.ac.uk/help/secure-lab/what-is-the-five-safes-framework/ [Accessed 13 December 2022].

19 Explore the Cancer Dependency Map (2021). https://depmap.org/portal/ [Accessed 13 December 2022].

20 Network Computing (2019). 3 Hidden Public Cloud Costs and How to Avoid Them. https://www.networkcomputing.com/cloud-infrastructure/3-hidden-public-cloud-costs-and-how-avoid-them [Accessed 16 August 2022].

21 World Health Organization (2022). WHO’s Science Council launches report calling for equitable expansion of genomics. https://www.who.int/news/item/12-07-2022-who-s-science-council-launches-report-calling-for-equitable-expansion-of-genomics. [Accessed 28 July 2022].
